# Synthesis and Biological Activity of New Brassinosteroid Analogs of Type 24-Nor-5β-Cholane and 23-Benzoate Function in the Side Chain

**DOI:** 10.3390/ijms22094808

**Published:** 2021-05-01

**Authors:** Nitza Soto, Karoll Ferrer, Katy Díaz, César González, Lautaro Taborga, Andrés F. Olea, Héctor Carrasco, Luis Espinoza

**Affiliations:** 1 Departamento de Química, Universidad Técnica Federico Santa María, Valparaíso, CP 2340000, Chile; nitza.soto@sansano.usm.cl (N.S.); karoll.ferrer.14@sansano.usm.cl (K.F.); katy.diaz@usm.cl (K.D.); cesar.gonzalez@usm.cl (C.G.); lautaro.taborga@usm.cl (L.T.); 2 Instituto de Ciencias Químicas Aplicadas, Facultad de Ingeniería, Universidad Autónoma de Chile, Santiago, CP 8900000, Chile; hector.carrasco@uautonoma.cl

**Keywords:** synthesis, brassinosteroids analogs, 24-nor-5β-cholane, 23-benzoates

## Abstract

Brassinosteroids are polyhydroxysteroids that are involved in different plants’ biological functions, such as growth, development and resistance to biotic and external stresses. Because of its low abundance in plants, much effort has been dedicated to the synthesis and characterization of brassinosteroids analogs. Herein, we report the synthesis of brassinosteroid 24-nor-5β-cholane type analogs with 23-benzoate function and 22,23-benzoate groups. The synthesis was accomplished with high reaction yields in a four-step synthesis route and using hyodeoxycholic acid as starting material. All synthesized analogs were tested using the rice lamina inclination test to assess their growth-promoting activity and compare it with those obtained for brassinolide, which was used as a positive control. The results indicate that the diasteroisomeric mixture of monobenzoylated derivatives exhibit the highest activity at the lowest tested concentrations (1 × 10^−8^ and 1 × 10^−7^ M), being even more active than brassinolide. Therefore, a simple synthetic procedure with high reaction yields that use a very accessible starting material provides brassinosteroid synthetic analogs with promising effects on plant growth. This exploratory study suggests that brassinosteroid analogs with similar chemical structures could be a good alternative to natural brassinosteroids.

## 1. Introduction

**Figure 1:**
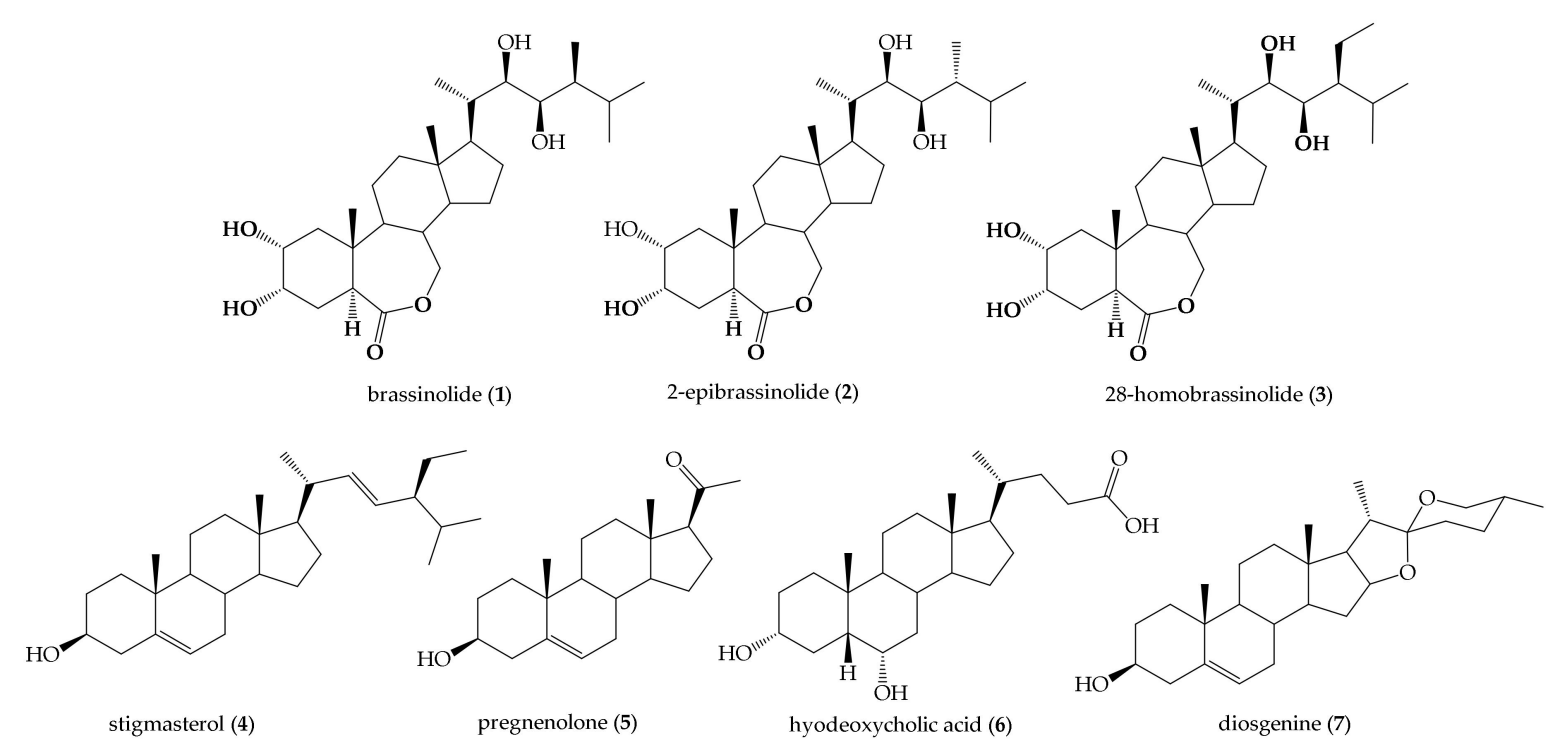
Structure of naturally occurring brassinosteroids and precursors.

Brassinosteroids (BRs) are phytohormones that are found in all plants; they act as plant growth promoters and development regulators at nanomolar to micromolar concentrations [1,2,3,4]. These phytohormones play important roles in cell development and vascular differentiation [5,6]. BRs are also involved in reproductive development, the modulation of gene expression [7,8] and other developmental processes like germination, rhizogenesis, flowering, senescence, abscission and maturation. Additionally, it has been proven that BRs induce plants’ resistance against different abiotic and biotic stresses [9,10,11]. All-natural bioactive BRs possess a vicinal 22*R*, 23*R* diol structural functionality (for example brassinolide **1**, 24-epibrassinolide **2** and 28-homobrassinolide **3**, Figure 1), which seems to be essential for high biological activity. In this sense, several studies have been focused on determining the structural requirements that these compounds should possess to elicit strong biological activity [12,13,14]. Structural variations of BRs principally arise from the type and position of functions in the A, B rings, fusion A/B ring and alkyl side chain.

The extremely low abundance of BRs present in plants (1–100 µg by kg of fresh weight) [15] has prompted the chemical synthesis of BR analogs, and a considerable number of them have already been reported [16]. However, to synthesize molecules with the same stereochemical configuration of natural BRs, both in the rings and side alkyl chain is an enormous synthetic challenge [17]. Consequently, synthetic BRs and BR analogs are generally too expensive for large-scale commercial applications. Therefore, these difficulties have stimulated the search for more accessible and bioactive analogs. The idea is to use natural molecules with chemical structures similar to BRs, such as stigmasterol (**4**) [18,19,20], pregnenolone (**5**) [21], hyodeoxycholic acid (**6**) [22,23] and diosgenin (7) [24,25] as starting material (Figure 1).

Hyodeoxycholic acid (**6**) is a bile acid extracted from hog bile whose chemical structure contains a similar ring skeleton and a side alkyl chain in the same position as BRs (see Figure 1). Considering that these structural features satisfy the requirements established for natural active BRs, brassinolide and several related compounds have been synthesized by chemical modification of **6** [26,27,28,29,30].

It is worth to mention that natural occurring BRs possess *trans*-type A/B ring fusion (5α BRs), and this structural factor has been proposed as a key factor for activity. However, studies of synthetic BRs analogs, having *cis*-type A/B ring fusion (5β BRs), have shown that these compounds exhibit important biological activity in tests such as rice lamina inclination (RLIT) [31], cotyledon expansion and hypocotile elongation radish [32,33,34] and recently in germination effects in tomato [35]. This shows that this structural characteristic of *cis* A/B ring fusion (5β skeleton) could be considered an alternative for new active BR analogs.

**Figure 2:**
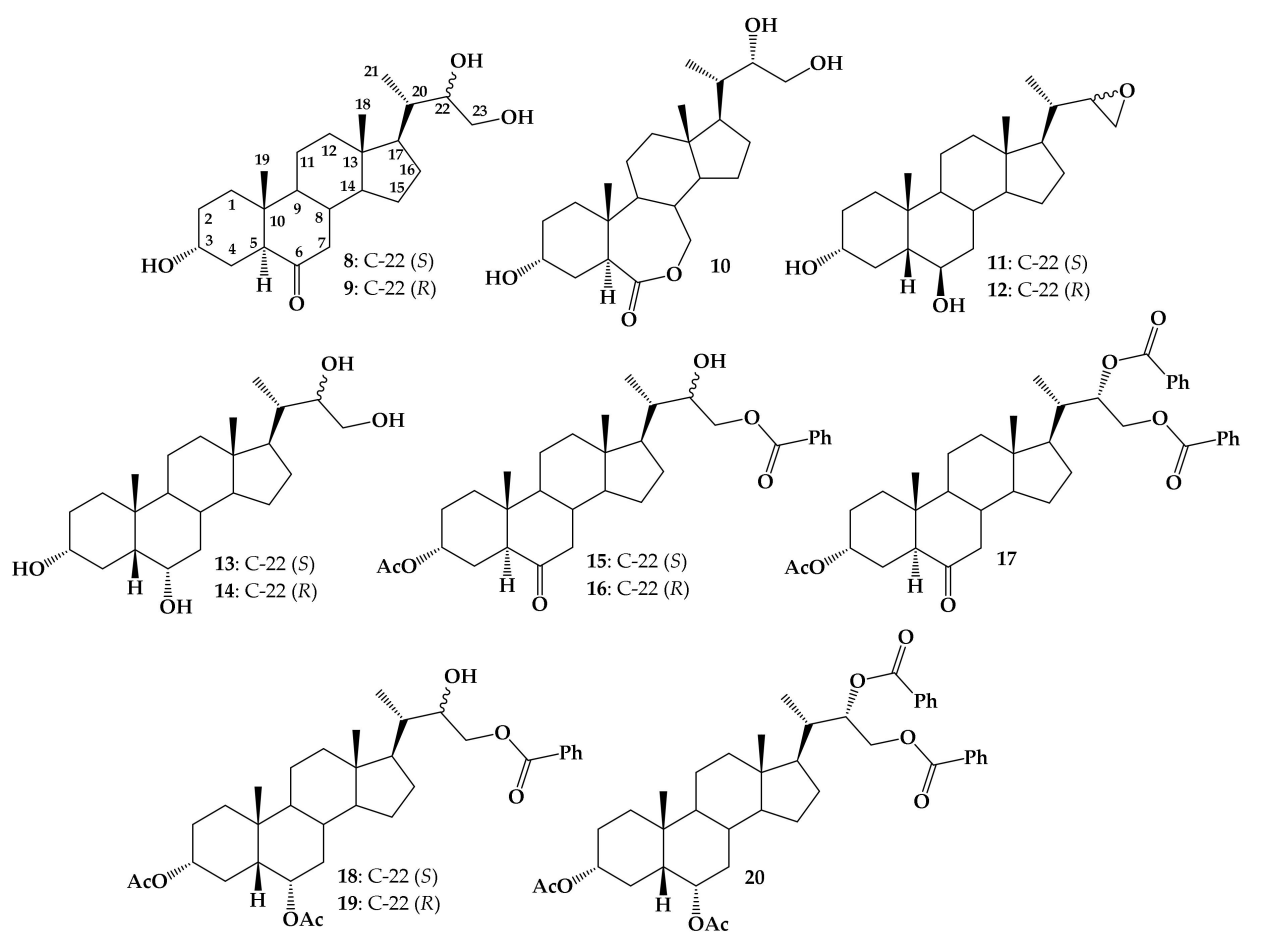
Structure of synthetic BRs 24-norcholan type **8**–**14** and 24-norcholan type **15**–**20** benzoylated analogs.

On the other hand, a series of new BR 24-nor-5α-cholane type analogs with a shortest side alkyl chain and an ending group in the form of carboxylic acids, methyl and phenyl esters (Figure 2) have also been synthesized from **6** [33,36,37,38]. Recently, the synthesis of BR analogs with a shortest side alkyl chain of 24-nor-5α-cholane type, oxygenated function in C22 with S/R configuration (Figure 2, compounds **8**–**14**) [39] and a benzoyl ester group at the ending chain (Figure 2, compounds **15**–**17**) were reported [38,39,40].

All these BR analogs were tested for bioactivity using the RLIT and using brassinolide (**1**) as positive control [35,40]. The results show that analog **8** (C22(*S*) configuration) was more active than diasteroisomer **9** (C22(*R*) configuration) and slightly less active than **1**. Interestingly, the activities of analogs with benzoyl groups in the side chain (**15** and **16**) do not depend on the C22 configuration, and both epimers show similar growth-promoting activities, which are almost identical to those exhibited by **1**. A molecular docking study suggest that this effect is due to hydrophobic interactions between the ligand and the receptor [40]. As a result, we have suggested that epimeric mixtures of *S* and *R* stereoisomers of monobenzoylated derivatives could be used instead of pure compounds [35].

Considering these results, in this work we have synthesized new BR 24-nor-5β-cholane type analogs with A/B ring fusion and acetate function in C6. Even though these analogs are similar to **15**, **16** and **17**, the synthesis of BR analogs with *cis* fusion of A/B rings requires fewer synthesis steps (isomerization reaction to convert fusion 5β to fusion 5α of A/B rings is no longer needed) and this is a clear synthetic advantage. In addition, it becomes interesting to evaluate the effect of this structural feature on the biological activity of these new compounds.

## 2. Results

The synthesis of BR analogs from hyodeoxycholic acid goes through the formation of a terminal olefin.

### 2.1. Chemistry

**Scheme 1:**
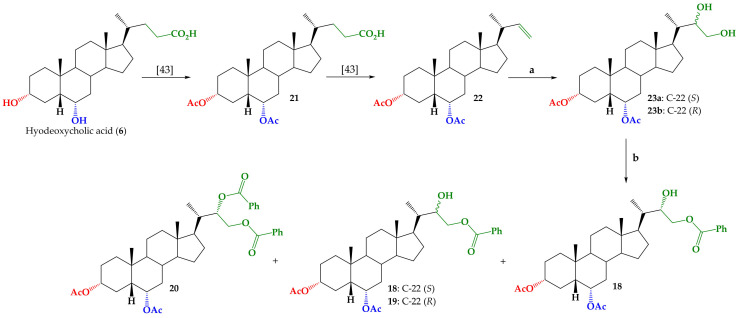
Synthesis of BR 24-nor-5β-cholane type analogs with function 23-benzoate **18**–**20**. Reagents and conditions: first two steps are described in Ref. [43] (**a**) DHQD-CLB/CH_3_SO_2_NH_2_, K_2_CO_3_/K3[Fe(CN)_6_], OsO_4_/(CH_3_)_3_COH/H_2_O, r.t, 1.5 h **23a**/**23b** (1.0:0.7), 95% yield; (**b**) C_6_H_5_COCl/DMAP/CH_2_Cl_2_/py, r.t, 36 h, **18** (11.3% yield), **18**/**19** (1.0:0.44, 78.2% yield), **20** (9.1% yield).

Standard acetylation of **6** (Scheme 1) leads to known diacetylated derivative **21** with 91.1% yield (referential: 80% yield [41,42,43]). Oxidative decarboxylation of the side chain of **21**, with the PhI(OAc)_2_/Cu(OAc)_2_ system [41,42], leads to alkene **22** with 99.6% yield (Scheme 1).

**Figure 3:**
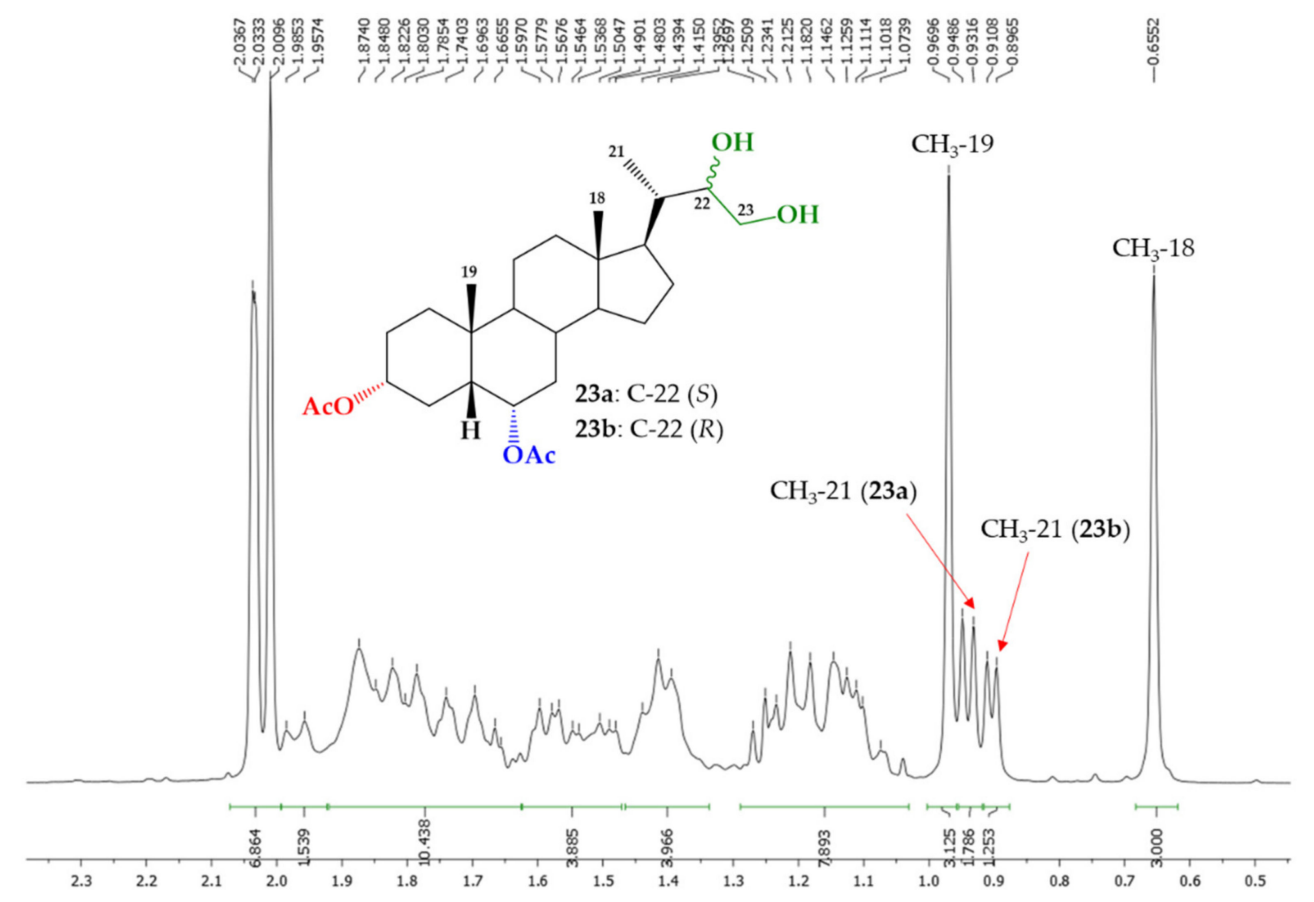
Amplification zone 0.5–2.2 ppm of ^1^H NMR of diasteroisomeric mixture **23a**/**23b**.

Previously, the synthesis of glycols C22/C23 in steroids of 24-nor-5α-cholane type with shortest side chain (terminal olefins) have been accomplished by Sharpless dihydroxylation reaction [38]. Results indicate that this type of hydroxylation leads to a mixture of C-22 glycols (*S*/*R*) with an approximate ratio 1.0:0.9 of both diastereomers, the 22*S* epimer being the slightly major product [38]. Thus, compound **22** was dihydroxylated by Sharpless reaction and using dihydroquinidine *p*-chlorobenzoate (DHQD-CLB) as chiral ligand (Scheme 1) [38,44]. The product of this reaction is the **23ab** diastereoisomer mixture with a total 95.0% yield. Separation of the **23ab** diastereoisomer mixture was not possible, but some ^1^H signals and all ^13^C signals in the NMR spectra of the mixture were identified. Thus, it was possible to determine that the C21 methyl group, in **23a** and **23b** diastereoisomers, appears at *δ*_H_ = 0.94 and 0.90 ppm, respectively (Figure 3). Integration of these signals gives a relative ratio of **23a**:**23b** equal to 1.0:0.7.

Additionally, the structure of compounds **23a** and **23b** and stereochemistry at C22 was established by simple comparison of ^1^H and ^13^C NMR spectra obtained for other 22,23-dihydroxy analogs of similar structure and the 24-norcholan type previously reported [38,39]. Thus, chemical shifts (*δ*), coupling constants (*J*) and multiplicities of signals appearing in ^1^H NMR, and chemical shifts (*δ*) in ^13^C NMR spectra, corresponding to H-22, H-23a, H-23b and CH_3_-21, were compared for both diasteroisomers. The main differences in these spectroscopic parameters were observed between H-21 and carbons C21, C22 and C23 (Figure S1, Supplementary Materials), and they are listed in Table 1.

**Figure 4:**
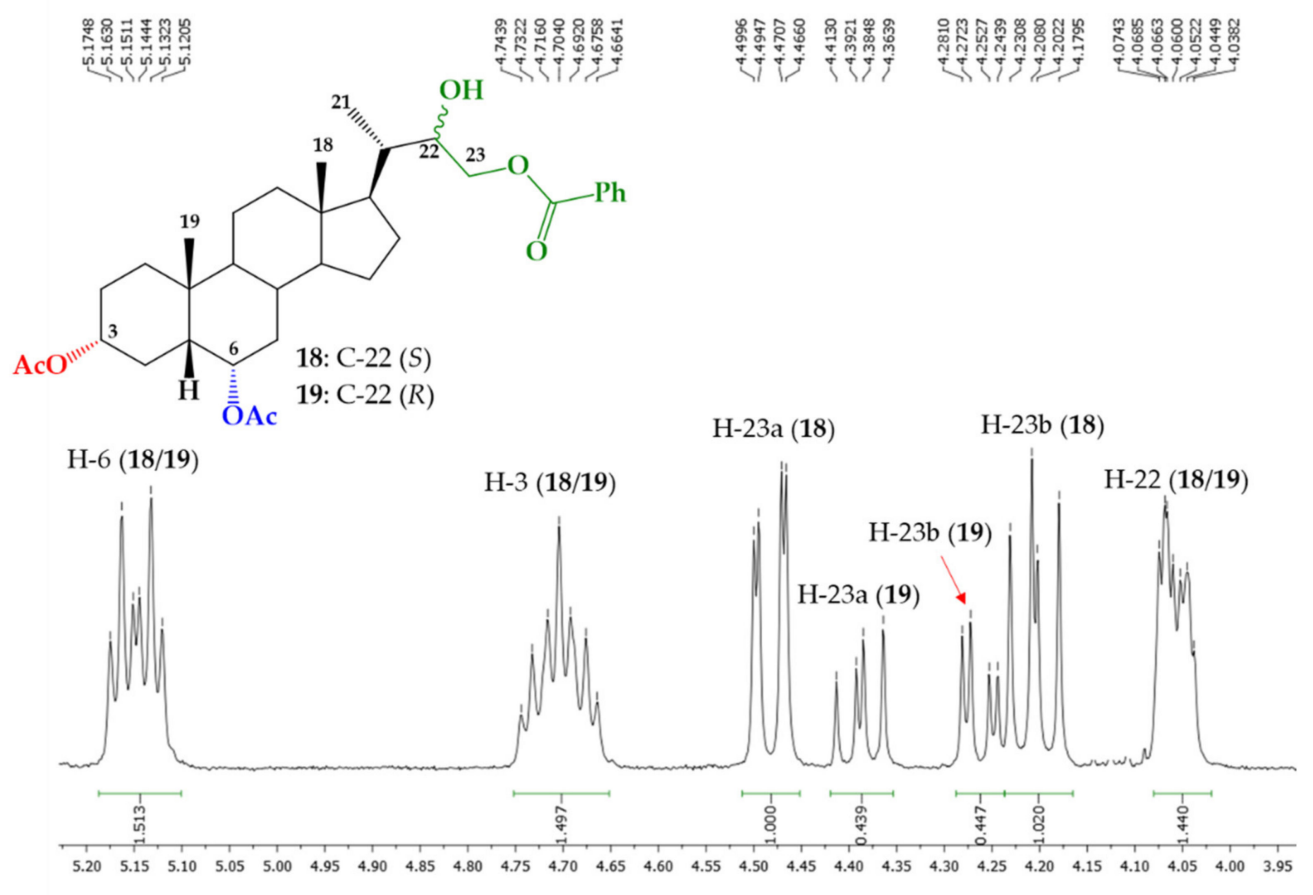
Amplification of 1H NMR spectrum of mixture **18**/**19** in the zone 3.95–5.20 ppm.

Benzoylation of glycol mixtures **23a**/**23b** gives mono- and dibenzoylated derivatives, i.e., compounds **18**-**20** (Scheme 1). Compounds **18** and **20** were purified by C.C. and recrystallized, giving yields of 11.3% and 9.1%, respectively, whereas monobenzoylated derivative **19** could not be isolated in pure form. Additionally, a mixture of monobenzoylated epimers **18**/**19** were also obtained with 78.2% yield (**18**:**19** = 1.0:0.44). The proportion of each epimer in the mixture was determined from integration of ^1^H NMR signals at *δ*_H_ = 4.48, 4.39, 4.26 and 4.05 ppm, assigned to H-23a, H-23b and H-22 for **18** and **19** (Figure 4).

The structure of compounds **18** and **19** and stereochemistry at C22 in the mixture was established in a way similar to that used for the mixture **23a**/**23b**, i.e., a simple comparison of ^1^H and ^13^C NMR spectra with those previously reported for other 22-hydroxy-23-benzoate and 24-norcholan type [38,39]. In addition, the comparison of ^1^H NMR spectra of pure compound **18** and the mixture **18**/**19** allows the assignment of signals for each compound (Figures S2 and S3, Supplementary Materials). A comparison of NMR spectroscopic parameters corresponding to signals assigned to H-22, H-23a, H-23b and CH_3_-21 in both diasteroisomers is given in Table 2. The main differences in these spectroscopic parameters were observed between H-21 and carbons C21, C22 and C23.

**Figure 5:**
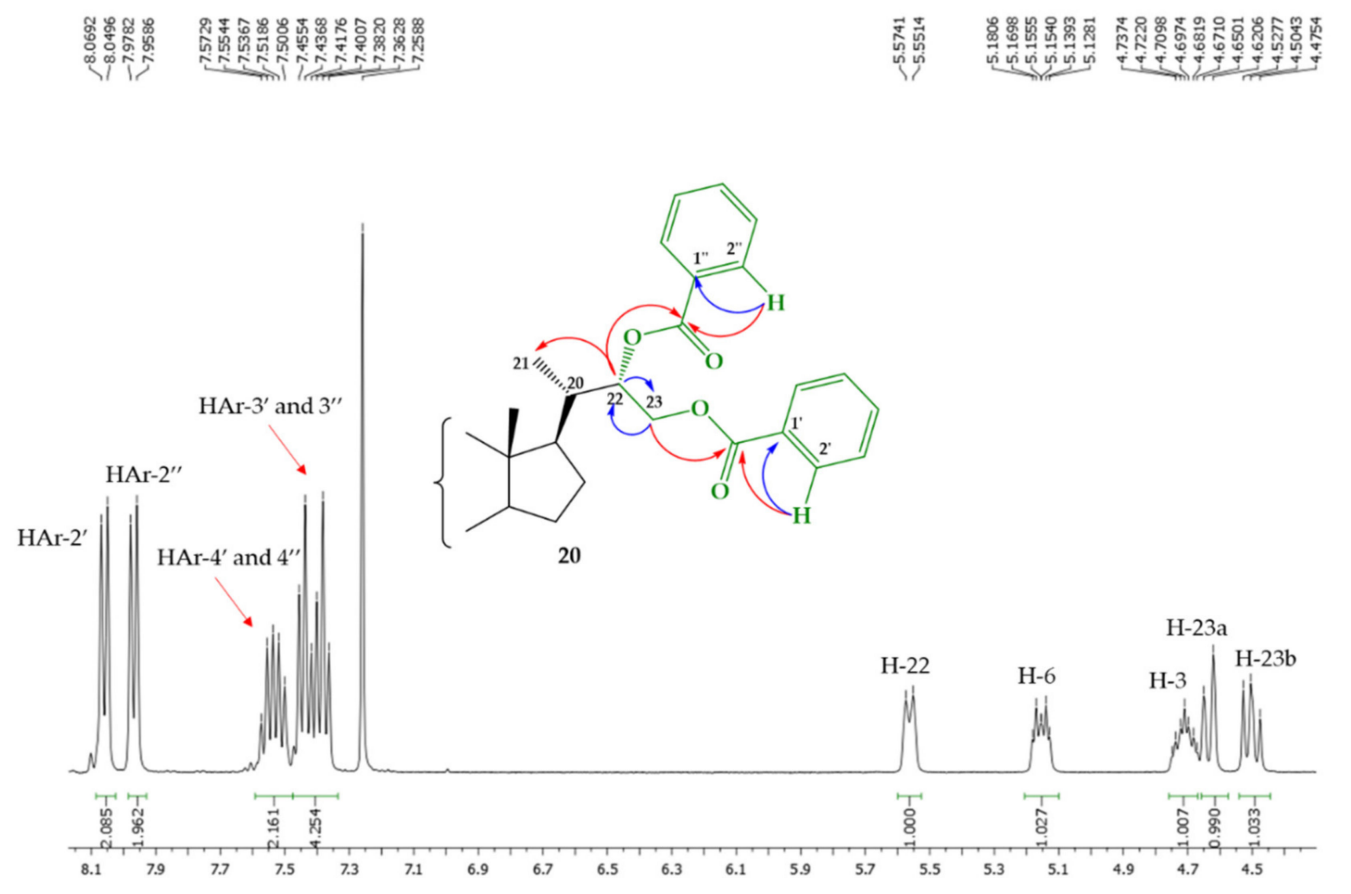
Amplification zone 8.15–4.45 ppm of ^1^H NMR for dibenzoylated derivative **20** and the main heteronuclear correlations 2D HMBC to ^2^*J*_CH_ (blue) and ^3^*J*_CH_ (red) observed for **20**, which confirm the positions of both benzoate groups in C22 and C23 of the side chain. HAr-2′; HAr-2″, HAr-4′, and HAr-4″; HAr-3′ and HAr-3″ are hydrogen atoms in the aromatic rings

The chemical structure of dibenzoylated derivative **20** was established using ^1^H, ^13^C, 2D HMQC, and 2D HMBC NMR techniques. The presence of dibenzoylated function was confirmed by signals observed in the ^1^H-NMR spectrum (Figure S4, Supplementary Materials), at *δ*_H_ = 8.06 (2H, d, *J* = 7.8 Hz); 7.97 (2H, d, *J* = 7.8 Hz); 7.57-7.50 (2H, m); 7.44 (2H, t, *J* = 7.6 Hz); 7.38 (2H, t, *J* = 7.6 Hz), which are assigned to protons HAr-2′; HAr-2″, HAr-4′, and HAr-4″; HAr-3′ and HAr-3″ in the aromatic group, respectively (Figure 5). In addition, in the ^13^C-NMR spectrum (Figure S4, Supplementary Materials) appear signals at 133.04; 132.98; 132.96; 130.38; 129.81; 129.65; 129.62; 128.37 and 128.35 ppm, which were assigned to carbon C4′-Ar; C1′-Ar; C4″-Ar; C2′-Ar; C1″-Ar; C3′-Ar; C2″-Ar; C3″-Ar and C4′-Ar, respectively, of both aromatic rings. These assignments were confirmed by 2D HMQC and HMBC spectra (Figure S4, Supplementary Materials). The main structural information provided by the 2D HMBC spectrum was the assignment and position in the side chain of the two benzoate groups, which showed heteronuclear correlations at ^2^*J*_CH_ and ^3^*J*_CH_, as indicated in Figure 5.

To determine the stereochemistry at carbon C22 of compound **20**, the monobenzoylated derivative **18** was submitted to an exhaustive benzoylation reaction with PhCOCl/DMAP. A dibenzoylated derivative was obtained with 93% yield, for which NMR data were totally consistent with those obtained for derivative **20**. Thus, we can conclude that analogs **18** and **20** have the same configuration at C22.

### 2.2. Bioactivity of BR Analogs Determined by Rice Lamina Inclination Assay (RLIT)

Since the discovery of BRs, it has been well-established that growth-promoting activity in plants is one of the most important functions in which natural BRs are involved [2,45]. Different bioassays have been developed and applied to detect and quantify this effect, i.e., first bean internode, root growth and RLIT. The latter is the most widely used test due to its high specificity and sensitivity, which allows for the detecttion of brassinolide concentrations at a level of 0.05 ng/L [46,47]. Using this test, the growth-promoting activity of a series of natural BRs and synthetic analogs have been evaluated. This data have been analyzed in terms of BR chemical structure, and different structure–activity relationships have been proposed [14,16,31]. In this work, the growth-promoting activities of **18**, diasteroisomeric mixture **18**/**19** and **20** were assessed by RLIT and using **1** as positive control. The reported bending angles (see Table 3) correspond to the mean ± standard deviation of two independent experiments with at least six replicates each (n = 12) for each tested concentration.

The results in Table 3 indicate, that at the lowest tested concentrations (1 × 10^−8^ and 1 × 10^−7^ M), the mixture of monobenzoylated compounds **18**/**19** exhibit activities even higher than that obtained for **1**, which is recognized as the most active natural BR. However, at the highest concentration (1 × 10^−6^ M), **1** becomes the most active once again. On the other hand, no significant differences in activity were observed between analog **18** and mixture **18**/**19** at concentrations of 1 × 10^−7^ and 1 × 10^−6^ M (Table 3). Considering that the ratio **18**:**19** is 1.0:0.44, this result suggests that, at these concentrations, the S epimer (**18**) is more active than the R epimer (**19**). However, at the lowest tested concentration, this behavior is reversed. Finally, the addition of a second benzoate group at the C22 position in the dibenzoylated analog **20** reduces biological activity significantly compared to analog **18** or epimeric mixture **18**/**19** (Table 3). These results are in line with those previously reported for similar BR 24-norcholane type analogs (compound **17**). Molecular docking studies of **15**, **16** and **17** have shown that decreasing activity with increasing size in substituent groups in the chain can be attributed to the lowest interaction between the side chain and the BRI1/BAK1 complex [40]. Thus, it seems that substitution in ring B by a carbonyl group (**15**, **16**, **17**) or an acetyl group (**18**, **19**, **20**) makes no difference to the growth enhancement effect of 24-norcholane type analogs.

## 3. Materials and Methods

### 3.1. Chemistry

#### 3.1.1. General Experimental Methods

All reagents were obtained from commercial suppliers at the highest quality grade available and used as received. Conditions and instruments used for measurement of melting points and recording of NMR, FT-IR, and MS spectra are given in detail in previous work [43]. Analytical TLC was run on silica gel 60 in a 0.25 mm layer, and TLC spots were detected by heating after spraying with diluted sulfuric acid (25%). Column chromatographic separations (CC) were carried out, using silica gel 60 (230–400 mesh) as stationary phase EtOAc-hexane gradients of increasing polarity as eluent. All organic extracts were dried over anhydrous magnesium sulfate and evaporated under reduced pressure, below 40 °C.

#### 3.1.2. Synthesis of Mixture (22S)-22,23-Dihydroxy-24-nor-5β-cholan-3α,6α-diyl Diacetate (**23a**) and (22R)-22,23-Dihydroxy-24-nor-5β-cholan-3α,6α-diyl Diacetate (**23b**)

Alquene **22** (250 mg, 0.58 mmol), DHQD-CLB (80 mg, 0.17 mmol), CH_3_SO_2_NH_2_ (120 mg, 1.22 mmol), K_2_CO_3_ (500 mg, 3.65 mmol) and K_3_[Fe(CN)_6_] (860 mg, 2.61 mmol) were successively added to a *t*-butanol/water mixture (22 mL, 1:1 *v*/*v*). After homogenization by magnetic stirring (5 min), 0.36 mL of an OsO_4_ solution (1.0 g, 3.933 mmol in 20 mL of *t*-butanol) was added, and the mixture reaction was stirred at room temperature for 1.5 h. After this period, H_2_O (10 mL) and a saturated solution of Na_2_S_2_O_3_^x^5H_2_O (10 mL) were added. The organic phase was extracted with AcOEt (2 ×x 30 mL), dried over MgSO_4_, filtered, and the solvent volume was reduced by evaporation under vacuum. The extract, dissolved in DCM (10 mL), was chromatographed on silica gel using hexane/EtOAc mixtures of increasing polarity (19.8:0.2 → 20.0:0.0). A mixture of **23a**/**23b** (257 mg, 95% yield, **23a**:**23b** = 1.0:0.7) was obtained as a colorless powder. IR ν_max_ (cm^−1^) mixture **23a**/**23b**: 3446 (O-H); 2934 (C-H); 2870 (C-H); 1737 (C=O); 1717 (C=O). **23a** ^1^H NMR (400.1 MHz, CDCl_3_) (Figure S1, Supplementary Materials): *δ* (ppm) 5.14 (1H, dt, *J* = 11.5 and 5.3 Hz, H-6); 4.70 (1H, m, H-3); 3.80 (1H, m, H-22); 3.63 (1H, dd, *J* = 9.9 and 9.9 Hz, H-23a); 3.51 (1H, m, H-23b); 2.04 (3H, s, CH_3_CO_2_-C6); 2,01 (3H, s, CH_3_CO_2_-C3); 0.97 (3H, s, H-19); 0.94 (3H, d, *J* = 6.8 Hz, H-21); 0.66 (3H, s, H-18). ^13^C NMR (Figure S3, Supplementary Materials) *δ* (ppm): 170.52 (CH_3_CO-C3); 170.49 (CH_3_CO-C6); 74.11 (C22); 73.64 (C3); 70.87 (C6); 62.47 (C23); 56.04 (C14); 55.78 (C17); 45.37 (C5 and C20); 42.72 (C13); 39.89 (C12); 36.04 (C10); 35.01 (C7); 34.68 (C8); 31.25 (C1); 27.78 (C2); 26.41 (C16); 26.22 (C4); 24.17 (C15); 24.02 (C19); 21.40 (CH_3_CO-C3); 21.37 (CH_3_CO-C6); 20.67 (C11); 12.62 (C21); 11.75 (C18). **23b** ^1^H NMR (Figure S1, Supplementary Materials) *δ* (ppm): 5.14 (1H, dt, *J* = 11.5 and 5.3 Hz, H-6); 4.70 (1H, m, H-3); 3.80 (1H, m, H-22); 3.63 (1H, dd, *J* = 9.9 and 9.9 Hz, H-23a); 3.51 (1H, m, H-23b); 2.04 (3H, s, CH_3_CO_2_-C6); 2.01 (3H, s, CH_3_CO_2_-C3); 0.97 (3H, s, H-19); 0.90 (3H, d, *J* = 5.7 Hz, H-21); 0.66 (3H, s, H-18). ^13^C NMR (Figure S1, Supplementary Materials) *δ* (ppm): 170.52 (CH_3_CO-C3); 170.49 (CH_3_CO-C6); 73.88 (C22); 73.64 (C3); 70.93 (C6); 66.04 (C23); 56.04 (C14); 55.78 (C17); 45.37 (C5 and C20); 45.32 (C9); 40.10 (C13); 39.82 (C12); 36.01 (C10); 35.01 (C7); 34.68 (C8); 31.25 (C1); 27.54 (C2); 26.41 (C16); 26.22 (C4); 24.17 (C15); 24.01 (C19); 21.40 (CH_3_CO-C3); 21.37 (CH_3_CO-C6); 20.67 (C11); 13.01 (C21); 11.87 (C18). MS *m*/*z* (%) (**23a**/**23b**): 464 (M^+^ < 1); 313 (25.6); 312 (100); 213 (21.7).

#### 3.1.3. Synthesis of (22S)-22-Hydroxy-24-nor-5β-cholan-3α,6α-diyl Diacetate-23-benzoate (**18**), (22R)-22-Hydroxy-24-nor-5β-cholan-3α,6α-diyl Diacetate-23-benzoate (**19**) and (22S)-24-nor-5β-cholan-3α,6α-diyl diacetate-22,23-diyl dibenzoate (**20**)

To a **23a**/**23b** mixture, (500 mg, 1.08 mmol) dissolved in CH_2_Cl_2_ (20 mL), were added DMAP (10 mg, 0.410 mmol) and pyridine (2.0 mL, d = 0.981 g/mL). The temperature was decreased to 0–5 °C under nitrogen atmosphere, and PhCOCl (1.4 mL, d = 1.21 g/mL, 12.05 mmol) was slowly added over 1 h. After addition, the reaction mixture was maintained at room temperature for 36 h. At the end of the reaction, the solvent volume was reduced by rotatory evaporation up to a final volume of 10 mL. The reaction mixture was diluted with EtOAc (40 mL), washed with a saturated solution of KHSO_4_ (2 × 10 mL) and H_2_O (3 × 30 mL), dried over MgSO_4_, and filtered. The solvent was evaporated under reduced pressure and the residue was redissolved in DCM (10 mL) and submitted to CC on silica gel with hexane/EtOAc mixtures of increasing polarity (19.8:0.2 -> 9.8:10.2). The obtained mixture (674.3 mg), formed by mono- and dibenzoylated compounds, was submitted to chromatographic separation twice. Three fractions were obtained in the following order of polarity: Fraction I compound **20** (65.7 mg, 9.1% yield); Fraction II mixture of compounds **18** and **19** (478.3 mg, **18**:**19** = 1.0:0.44, 78.2% yield); and Fraction III compound **18** (68.9 mg, 11.3% yield). Compound **18** was obtained as a colorless powder (m.p. 133 ± 3°C). IR ν_max_ (cm^−1^): 3523 (O-H); 2942 (C-H); 2890 (C-H); 2866 (C-O); 1735 (C=O); 1716 (C=O); 1276 (OCOAr). ^1^H NMR (400.1 MHz, CDCl_3_) (Figure S4, Supplementary Materials) *δ* (ppm): 8.05 (2H, d, *J* = 7.8 Hz, HAr-2′); 7.58 (1H, t, *J* = 7.3 Hz, HAr-4′); 7.45 (2H, t, *J* = 7.8 Hz, HAr-3′); 5.15 (1H, dt, *J* = 11.4 and 5.3 Hz, H-6); 4.70 (1H, m, H-3); 4.48 (1H, dd, *J* = 11.4 and 2.0 Hz, H-23a); 4.21 (1H, dd, *J* = 11.4 and 10.2, H-23b); 4.07-4.04 (1H, m, H-22); 2.02 (3H, s, 3H, CH_3_CO-C3); 2.01 (3H, s, CH_3_CO-C6); 1.04 (3H, d, *J* = 6.8 Hz, H-21); 0.98 (3H, s, H-19); 0.68 (3H, s, H-18). ^13^C NMR (Figure S2, Supplementary Materials) *δ* (ppm): 170.50 (CH_3_CO-C3); 170.45 (CH_3_CO-C3); 166.99 (CO-Ar); 133.17 (C4′-Ar); 129.90 (C1′-Ar); 129.62 (C2′-Ar); 128.42 (C3′-Ar); 73.63 (C3); 71.79 (C22); 70.87 (C6); 68.95 (C23); 55.78 (C17); 53.06 (C14); 45.34 (C9); 43.29 (C13); 40.34 (C20); 39.86 (C7); 39.81 (C5); 38.40 (C8); 36.03 (C10); 35.02 (C12); 31.26 (C1); 27.56 (C2); 26.42 (C4); 26.23 (C15); 24.18 (C16); 23.24 (C19); 21.40 (CH_3_CO-C3); 21.36 (CH_3_CO-C6); 20.66 (C11); 12.90 (C21); 11.80 (C18). MS *m*/*z* (%): 568 (M^+^ < 1); 327 (32.4); 326 (100); 213 (29.6); 207 (26.1); 145 (19.0); 133 (19.1). Compound **19** ^1^H NMR (400.1 MHz, CDCl_3_) (Figure S3, Supplementary Materials) *δ* (ppm): 8.05 (2H, d, *J* = 7.8 Hz, HAr-2′); 7.58 (1H, t, *J* = 7.3 Hz, HAr-4′); 7.45 (2H, t, *J* = 7.8 Hz, HAr-3′); 5.15 (1H, dt, *J* = 11.4 and 5.3 Hz, H-6); 4.70 (1H, m, H-3); 4.39 (1H, dd, *J* = 11.4 and 8.4 Hz, H-23a); 4.26 (1H, dd, *J* = 11.4 and 3.3 Hz, H-23b); 4.07-4.04 (1H, m, H-22); 2.05 (3H, s, CH_3_CO-C3), 2.02 (3H, s, CH_3_CO-C6); 1.00 (3H, d, *J* = 6.4 Hz, H-21); 0,98 (3H, s, H-19); 0.68 (3H, s, H-18). ^13^C RMN (Figure S3, Supplementary Materials) *δ*(ppm): 170.50 (CH_3_CO-C3); 170.45 (CH_3_CO-C3); 166,68 (CO-Ar); 133.08 (C4′-Ar); 129.88 (C1′-Ar); 129.62 (C2′-Ar); 128.36 (C3′-Ar); 73.63 (C3); 71.86 (C22); 70.87 (C6); 68.88 (C23); 55.78 (C17); 53.06 (C14); 45.34 (C9); 43.29 (C13); 40.34 (C20); 39.86 (C7); 39.81 (C5); 38.40 (C8); 36.03 (C10); 35.02 (C12); 31.26 (C1); 27.56 (C2); 26.42 (C4); 26.23 (C15); 24.18 (C16); 23.24 (C19); 21.40 (CH_3_CO-C3); 21.36 (CH_3_CO-C6); 20.66 (C11); 12.39 (C21); 11.90 (C18). Compound **20** was obtained as a colorless powder (m.p. 122 ± 3°C). IR ν_max_ (cm^−1^): 2946 (C-H); 2870 (C-H); 1282 (C-O); 1249 (C-O); 1723 (C=O); 1723 (C=O); 1283 (OCOAr); 1263 (OCOAr). ^1^H NMR (400.1 MHz, CDCl_3_) (Figure S4, Supplementary Materials) *δ*(ppm: 8.06 (2H, d, *J* = 7.8 Hz, HAr-2′); 7.97 (2H, d, *J* = 7.8 Hz, HAr-2″); 7.57-7.50 (2H, m, HAr-4′ and HAr-4″); 7.44 (2H, t, *J* = 7.6 Hz, HAr-3′); 7.38 (2H, t, *J* = 7.6 Hz, HAr-3″); 5.56 (1H, bd, *J* = 9.1 Hz, H-22); 5.16 (1H, dt, *J* = 11.4 and 5.3 Hz, H-6); 4.71 (1H, m, H-3); 4.64 (1H, d, *J* = 11.8 Hz, H-23a); 4.50 (1H, dd, *J* = 11.8 and 9.3 Hz, H-23b); 2.05 (3H, s, CH_3_CO-C3); 2.02 (3H, s, CH_3_CO-C6); 1.13 (3H, d, *J* = 6.0 Hz, H-21); 0.98 (3H, s, H-19); 0.69 (3H, s, H-18). ^13^C NMR (Figure S4, Supplementary Materials) *δ* (ppm): 170.50 (CH_3_CO-C3); 170.45 (CH_3_CO-C3); 166.61 (CO-Ar′); 165.91 (CO-Ar″); 133.04 (C4′-Ar); 132.98 (C1′-Ar); 132.96 (C4″-Ar); 130.38 (C2′-Ar); 129.81 (C1″-Ar); 129.65 (C3′-Ar); 129.62 (C2″-Ar); 128.37 (C3″-Ar); 128.35 (C4′-Ar); 74.67 (C22); 73.64 (C3); 70.87 (C6); 63.00 (C23); 55.82 (C15 and C17); 53.12 (C9); 45.35 (C5); 43.40 (C13); 39.86 (C7); 38.60 (C8); 36.03 (C10); 35.05 (C20); 34.67 (C12); 31.28 (C1); 27.41 (C2); 26.45 (C4); 26.25 (C15); 24.21 (C16); 23.25 (C19); 21.42 (CH_3_CO-C3); 21.37 (CH_3_CO-C6); 20.69 (C11); 13.76 (C21); 11.88 (C18). MS *m*/*z* (%): 673 (M^+^<1); 327 (26.0); 326 (100); 213 (33.3); 145 (22.2); 105 (20.4); 81 (19.1).

#### 3.1.4. Synthesis of (22S)-24-Nor-5β-cholan-3α,6α-diyl Diacetate-22,23-diyl Dibenzoate (**20**)

DMAP (5 mg, 0.205 mmol) and pyridine (0.5 mL, d = 0.981 g/mL) were successively added to a solution of **18** (25.0 mg, 0.044 mmol) in CH_2_Cl_2_ (10 mL) with stirring under nitrogen atmosphere. Then PhCOCl 0.5 mL (d = 1.21 g/mL, 4.30 mmol) was added, and the reaction mixture was maintained at room temperature for 3 h. At the reaction ending (verified by TLC), the solvent volume was taken to 2 mL by evaporation under reduced pressure. The resulting mixture was diluted with EtOAc (15 mL), washed successively with a saturated solution of KHSO_4_ (2 × 3 mL) and H_2_O (3 × 5 mL), dried over MgSO_4_, and filtered. The solvent was evaporated under reduced pressure. The crude was redissolved in DCM (5 mL) and purified by CC using hexane/EtOAc mixtures of increasing polarity (19.8:0.2 -> 9.8:10.2). Dibenzoylated compound **20** was obtained (24.7 mg) with 93% yield. The ^1^H and ^13^C-NMR spectroscopic data were consistent with those described above.

### 3.2. In Vitro Rice Lamina Inclination Test (RLIT)

The growth-promoting activity of all new synthesized BR analogs was evaluated, and compared with that shown by brassinolide, using the rice lamina inclination test. The experimental protocol has been described elsewhere [37,48]. Briefly, segments of rice leaf of approximately 6 cm were excised from growing rice plants that have reached a size of which the second internode can be obtained. Six of these segments per treatment were incubated for 48 h at 22 °C in the dark on Petri plates (50 mL) containing different concentrations (1 × 10^−8^ M, 1 × 10^−7^ M and 1 × 10^−6^ M) of testing samples (**18**, diasteroisomeric mixture **18**/**19** and **20**). Brassinolide (APE BIO, Boston, MA, USA) was used as a positive control, whereas pure sterile water was used as a negative control. After incubation, the opening angle formed between the leaf and the sheath was measured with a protractor. The reported magnitude of angle values are given as the mean of twelve measurements with standard deviations (n = 12). This test was performed twice for each treatment

## 4. Conclusions

The synthesis of BR 24-nor-5β-cholane type analogs with 23-benzoate function (**18**–**19**) and 22- and 23-benzoate groups (**20**) was accomplished with high reaction yields (18/19, 78%; 18, 11%; and 20, 9.1%), using hyodeoxycholic acid as starting material. All products and intermediate compounds were fully characterized, mainly by 1D and 2D NMR spectroscopic techniques.

Growth-promoting activity was assessed by RLIT and the results show that, at the lowest tested concentrations (1×10^−8^–1×10^−7^ M), the mixture **18**/**19** exhibit the highest activity, including higher than brassinolide. The high activity of similar 24-nor-5β-cholane type analogs carrying bulky groups at the end of the shortest side chain has been attributed to hydrophobic interaction between the aromatic ring with the BRI1/BAK1 complex [40]. In this case, the synthetic analogs are even simpler because the hydroxyl group in ring B of hyodeoxycholic acid was just acetylated instead of oxidized to a carbonyl group. In addition, the direct use of a diasteroisomers mixture avoids an arduous separation step. Thus, the results of growth-promoting activity exhibited by the mixture **18**/**19** along with its straightforward synthesis validate the synthetic approach described herein, as a potential method for the obtention of BR synthetic analogs with promising effects on plant growth.

## Figures and Tables

**Table 1:** Comparison between signals of ^1^H (400.1 MHz, CDCl_3_) and ^13^C (100.6 MHz, CDCl_3_) NMR for H/C21, H/C22 and H/C23a-b, for the diasteroisomers **23a** and **23b**.

H/C Signal	Compound 23a	Compound 23b
H-21	0.94 ppm (d, *J* = 6.8 Hz)	0.90 (d, *J* = 5.7 Hz)
H-22	3.81–3.79 ppm (m)	3.81–3.79 ppm (m)
H-23a	3.63 (dd, *J* = 9.9 and 9.9 Hz)	3.63 (dd, *J* = 9.9 and 9.9 Hz)
H-23b	3.51 (m)	3.51 (m)
C21	12.62 ppm	13.01 ppm
C22	74.11 ppm	73.88 ppm
C23	62.47 ppm	66.04 ppm

**Table 2:** Comparison between signals of ^1^H (400.1 MHz, CDCl_3_) and ^13^C (100.6 MHz, CDCl_3_) NMR for H/C21, H/C22 and H/C23a-b, for the diasteroisomers **18** and **19**.

H/C Signal	Compound 18	Compound 19
H-21	1.04 ppm (d, *J* = 6.8 Hz)	1.00 (d, *J* = 6.4 Hz)
H-22	4.07-4.04 ppm (m)	4.07–4.04 ppm (m)
H-23a	4.48 (dd, *J* = 11.4 and 2.0 Hz)	4.39 (dd, *J* = 11.4 and 8.4 Hz)
H-23b	4.21 (dd, *J* = 11.4 and 10.2 Hz)	4.26 (dd, *J* = 11.4 and 3.3 Hz)
C21	12.90 ppm	12.39 ppm
C22	71.79 ppm	71.86 ppm
C23	68.95 ppm	68.88 ppm

**Table 3:** Effect of compound **18**, epimeric mixture **18**/**19** and compound **20** on lamina inclination of rice seedlings.

Bending Angle between Lamina and Sheaths (Degrees ± Standard Error)				
Compound	Structure	Concentration [M]		
		1 × 10^−8^	1 × 10^−7^	1 × 10^−6^
**18**	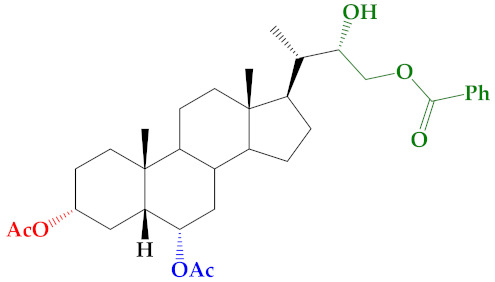	20 ± 1.0	48 ± 2.9	49 ± 2.5
**18**/**19** (1.0:0.44)	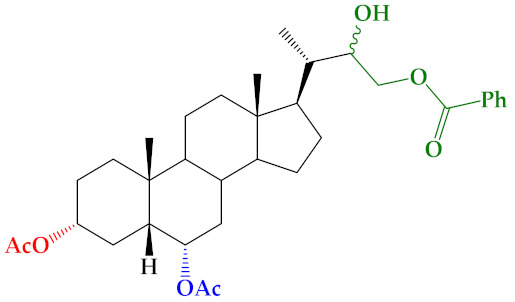	68 ± 2.5	50 ± 2.5	46 ± 2.9
**20**	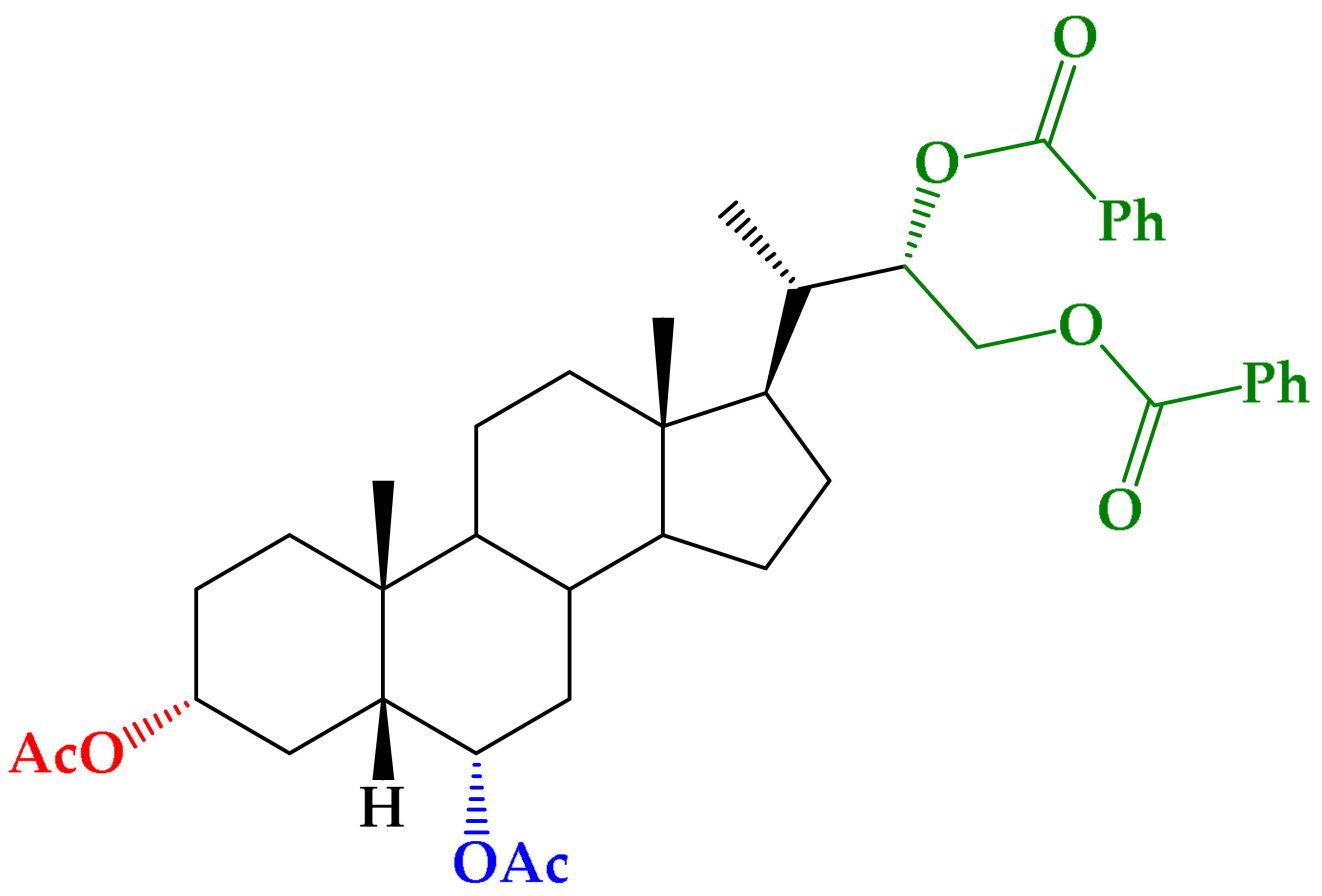	6 ± 2.5	25 ± 1.0	23 ± 2.1
**1** (C+) *	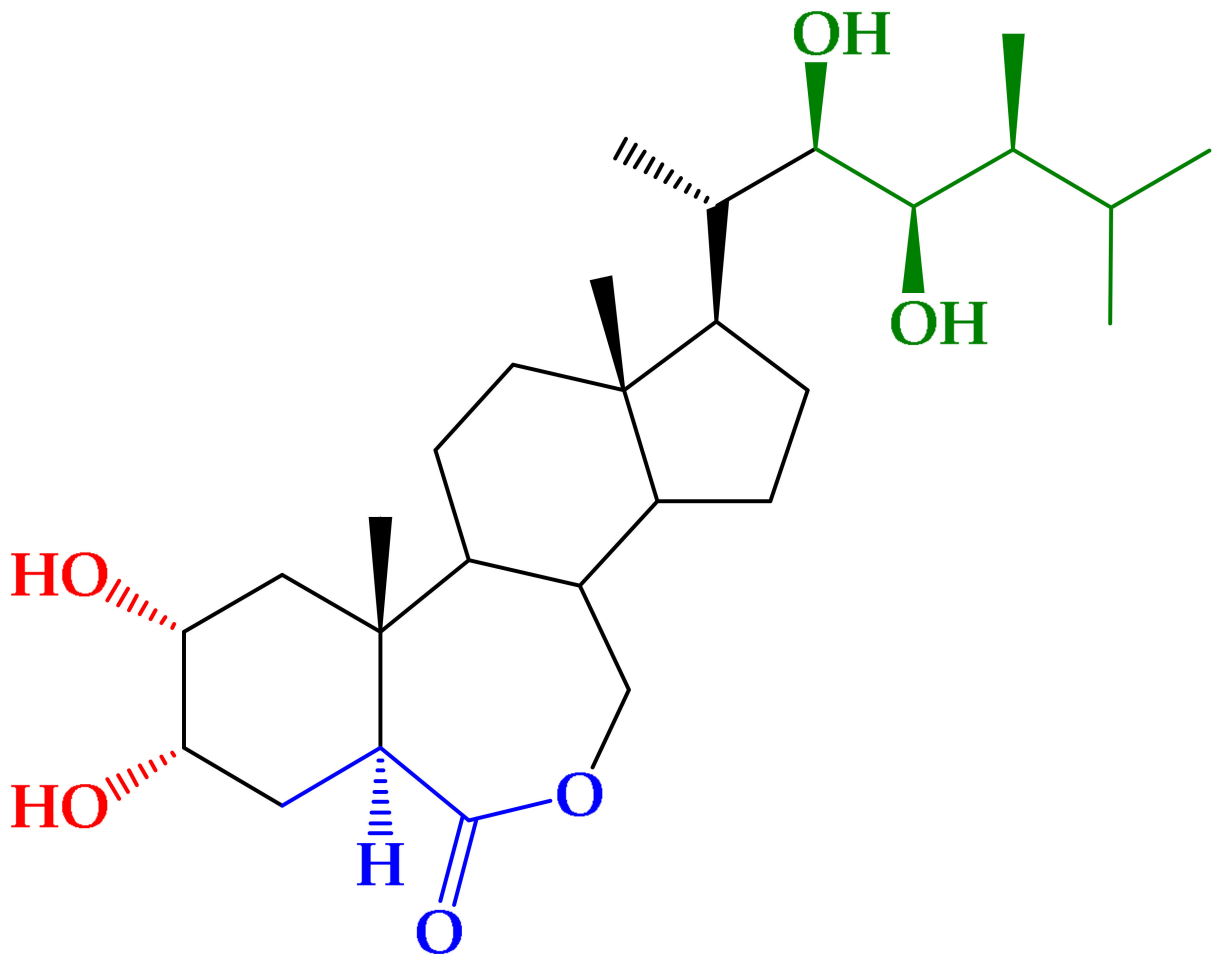	31 ± 1.1	41 ± 4.5	70 ± 7.6
Water (C−)		7 ± 4.5		

* 1 was used as positive control (C+).

## Data Availability

Not applicable.

## Supplementary Materials

The following are available online at https://www.mdpi.com/article/10.3390/ijms22094808/s1.
