# Supplementary material for: Synthesis and Biological Activity of New Brassinosteroid Analogs of Type 24-Nor-5β-Cholane and 23-Benzoate Function in the Side Chain
Source: Int J Mol Sci. 2021 May 1;22(9):4808. doi: 10.3390/ijms22094808 (PMC8124218; doi:10.3390/ijms22094808)
Supplement: Supplementary file 1 [file ijms-22-04808-s001.zip › ijms-1196643 supp.pdf]

International Journal of *Molecular Sciences*

MDPI

Type of the Paper (Article)

Synthesis and biological activity of new brassinosteroid analogs of type 24-nor-5β-cholane and 23-benzoate function in the side chain

Nitza Soto [1], Karoll Ferrer [1], Katy Díaz [1], César González [1], Lautaro Taborga [1], Andrés F. Olea [2]*, Héctor Carrasco [2] and Luis Espinoza [1,*]

[1] Universidad Técnica Federico Santa María, Departamento de Química, Av. España 1680, Valparaíso, Chile. CP 2340000; nitza.soto@sansano.usm.cl; karoll.ferrer.14@sansano.usm.cl; katy.diaz@usm.cl; cesar.gonzalez@usm.cl; lautaro.taborga@usm.cl; luis.espinozac@usm.cl

[2] Instituto de Ciencias Químicas Aplicadas, Facultad de Ingeniería, Universidad Autónoma de Chile, El Llano Subercaseaux 2801, Santiago, Chile and CP 8900000; hector.carrasco@uautonoma.cl; andres.olea@uautonoma.cl

* Correspondence: luis.espinozac@usm.cl; andres.olea@uautonoma.cl; Tel.: +56-32-2654425 (L.E.))

Supplementary Materials: **The following are available online at www.mdpi.com/xxx/s1, Figure S1**: NMR spectra of (22*S*)-22,23-dihydroxy-24-nor-5βcholan-33α,6α--diyl diacetate (**23a**) and (22*R*)-22,23-dihydroxy-24-nor-5β-cholan-3α,6α-diyl diacetate (**23b**), **Figure S2**: NMR spectra of (22*S*)-22-hydroxy-24-nor-5β-cholan-3α,6α-diyl diacetate-23-benzoate (**18**), **Figure S3**: NMR spectra of (22*S*)-22-hydroxy-24-nor-5β-cholan-3α,6α-diyl diacetate-23-benzoate (**18**) and (22*R*)-22-hydroxy-24-nor-5β-cholan-3α,6α-diyl diacetate-23-benzoate (**19**), **Figure S4**: NMR spectra of (22*S*)-24-nor-5β-cholan-3α,6α-diyl diacetate-22,23-diyl dibenzoate (**20**)

Citation: Soto, N.; Ferrer, K.; Díaz, K.; González, C.; Taborga, L.; Olea, A.F.; Carrasco, H.; Espinoza, L. Synthesis and biological activity of new brassinosteroid analogs of type 24-nor-5β-cholane and 23-benzoate function in the side chain. **2021**, *22*, 4808. https://doi.org/10.3390/ijms22094808

Received: date
Accepted: date
Published: date

Publisher's Note: MDPI stays neutral with regard to jurisdictional claims in published maps and institutional affiliations.

Copyright: © 2021 by the authors. Submitted for possible open access publication under the terms and conditions of the Creative Commons Attribution (CC BY) license (http://creativecommons.org/licenses/by/4.0/).

^{1}H-NMR

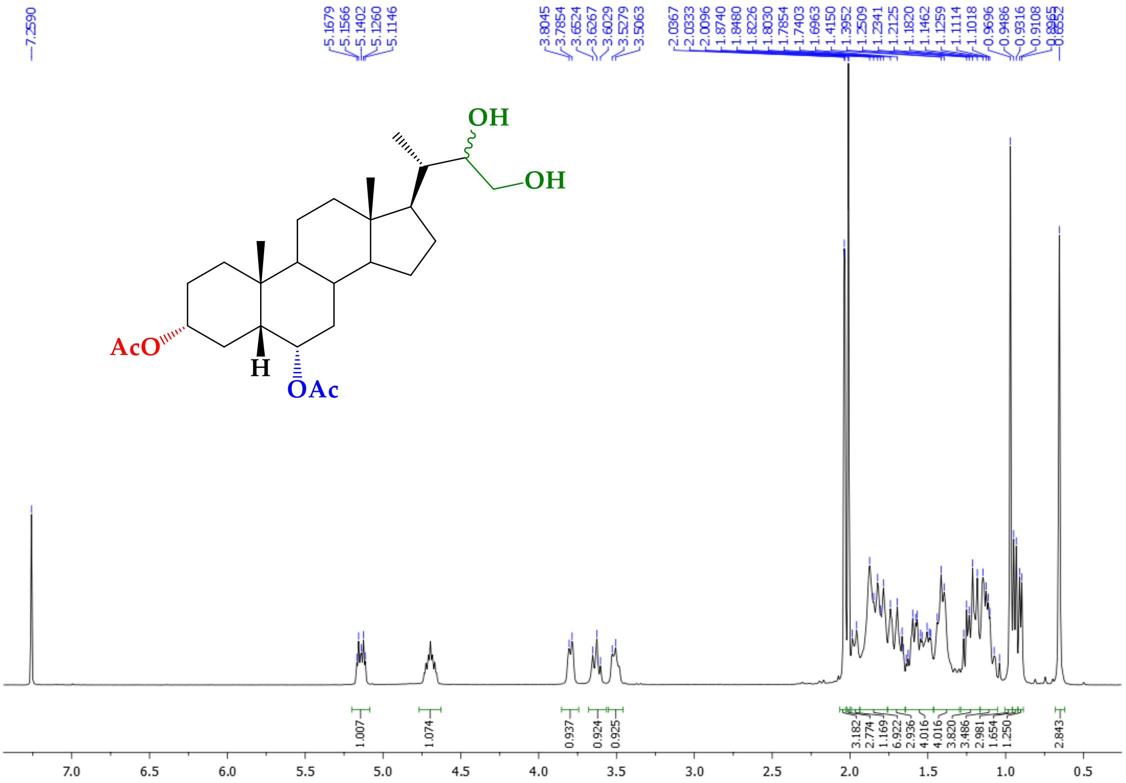

^{13}C-NMR

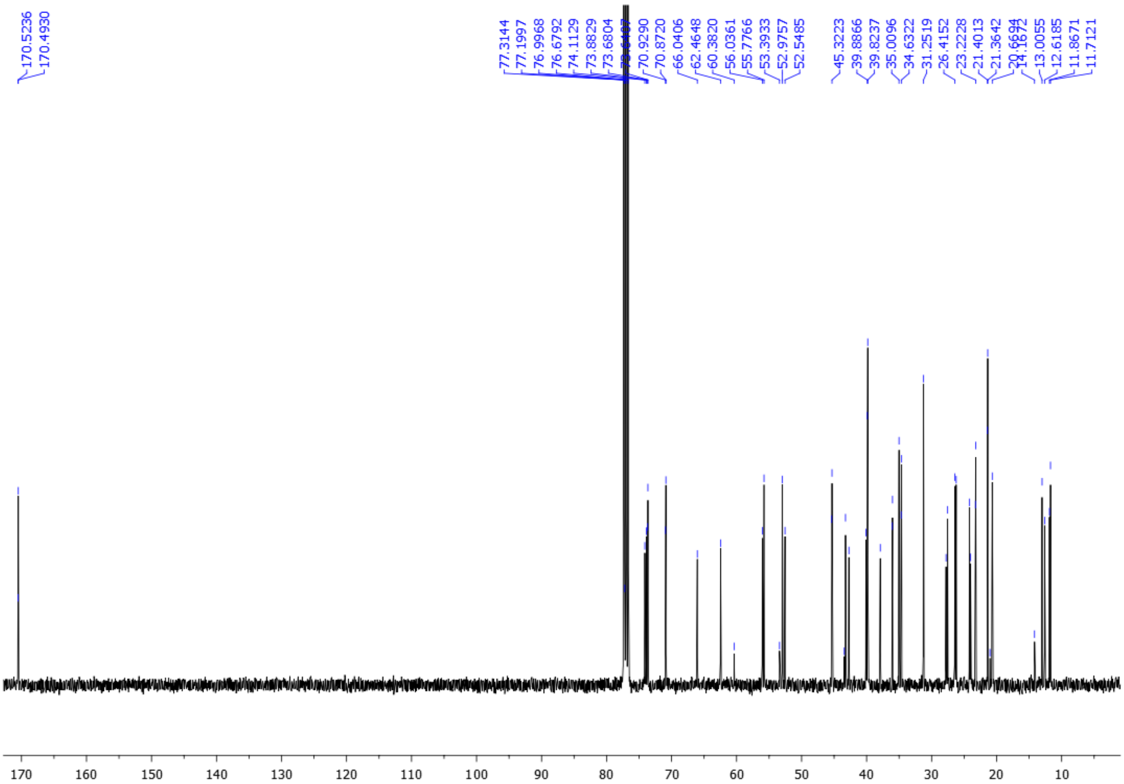

Figure S1: NMR spectra of (22*S*)-22,23-dihydroxy-24-nor-5βcholan-33α,6α--diyl diacetate (**23a**) and (22*R*)-22,23-dihydroxy-24-nor-5β-cholan-3α,6α-diyl diacetate (**23b**)

¹H-NMR

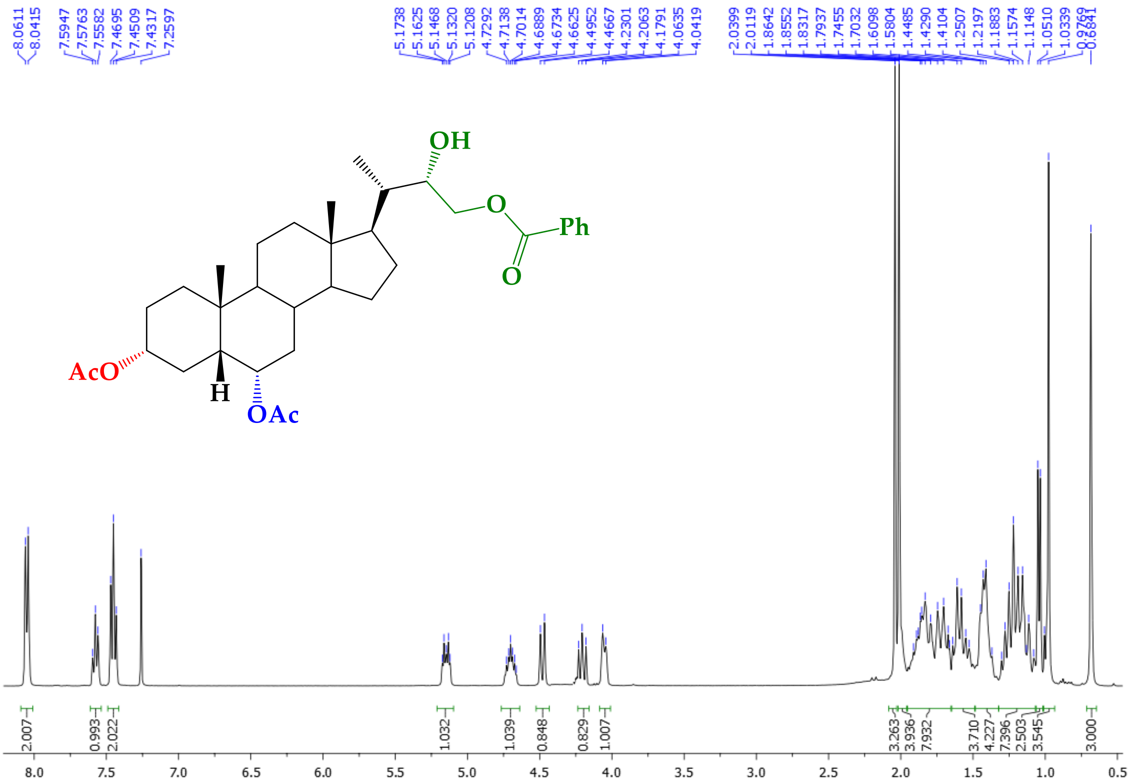

¹³C-NMR

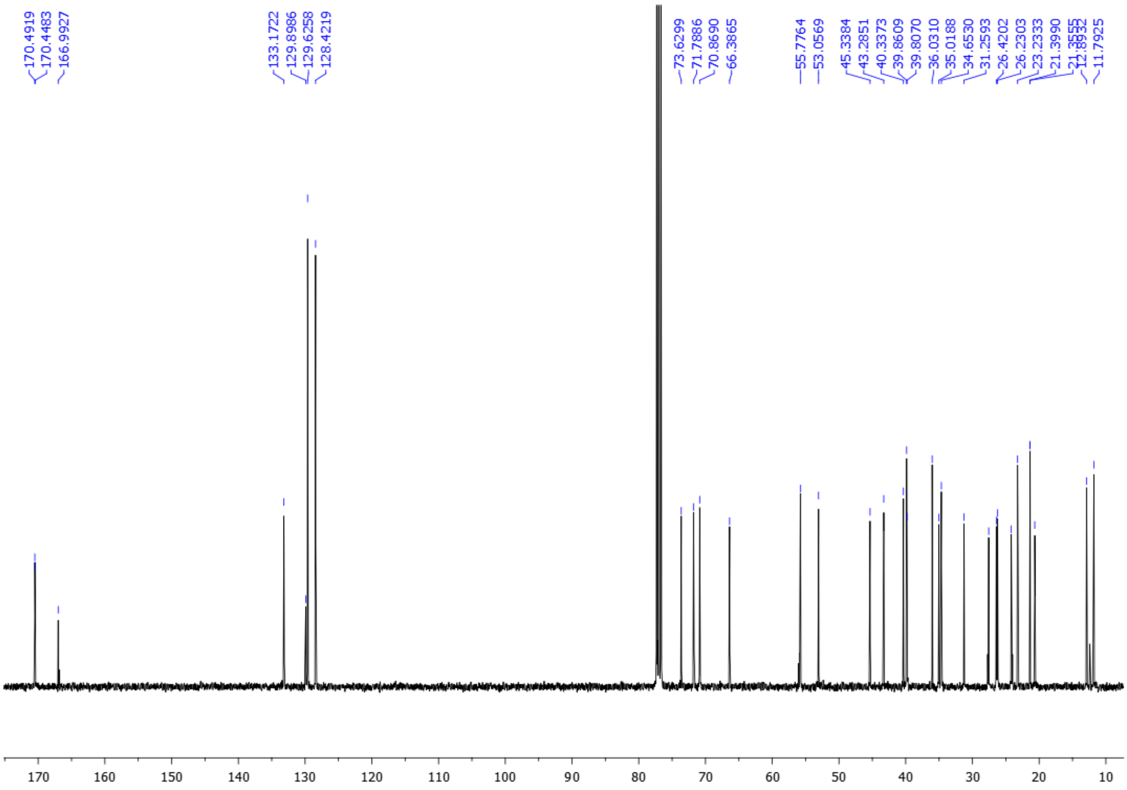

Figure S2: NMR spectra of (22*S*)-22-hydroxy-24-nor-5β-cholan-3α,6α-diyl diacetate-23-benzoate (**18**).

^{1}H-NMR

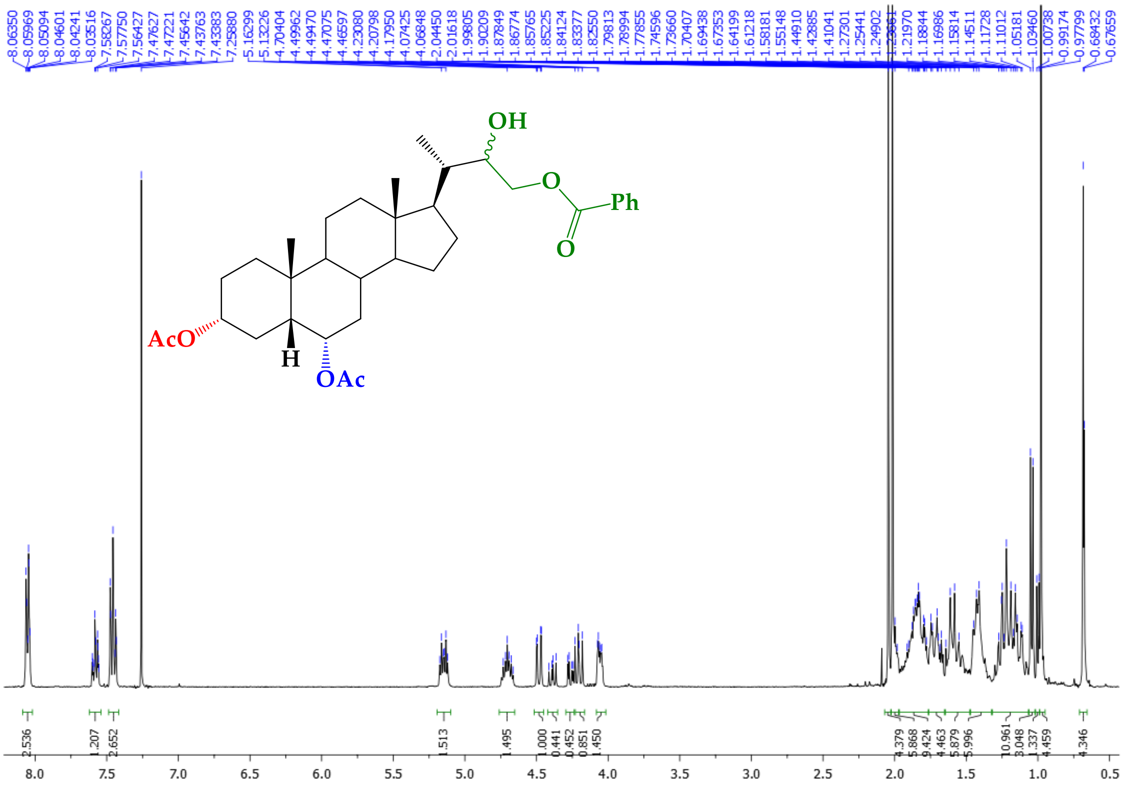

^{13}C-NMR

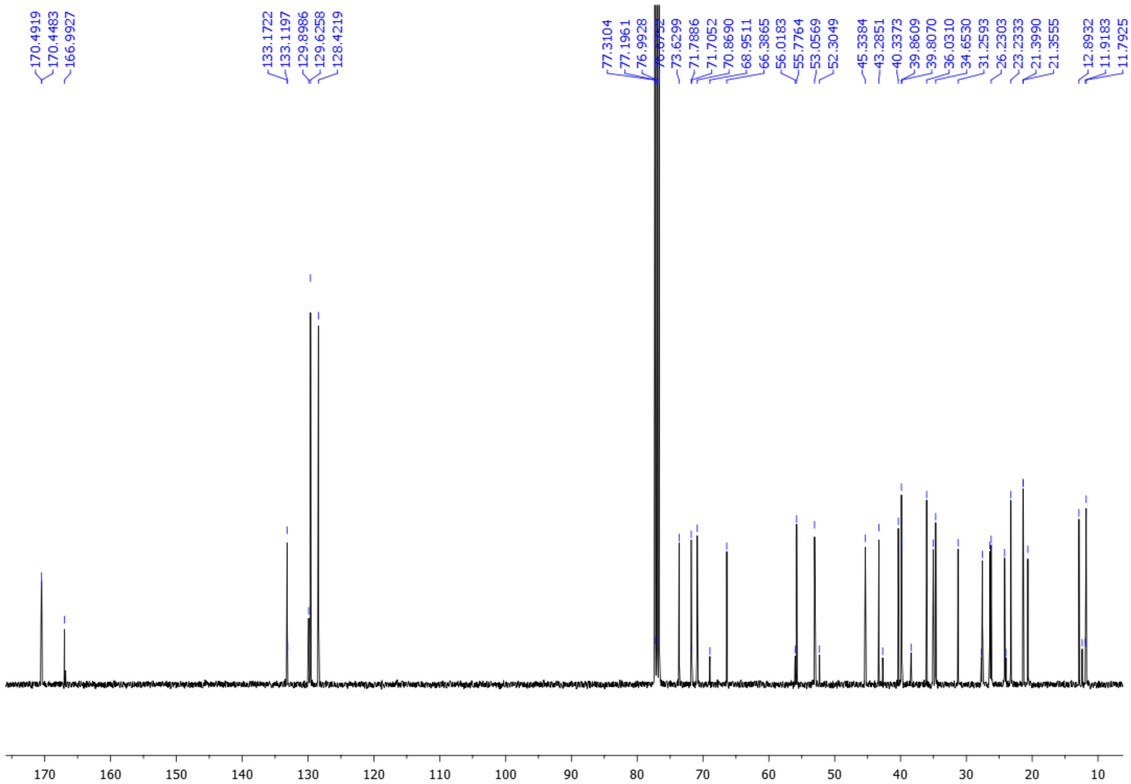

Figure S3: NMR spectra of (22*S*)-22-hydroxy-24-nor-5β-cholan-3α,6α-diyl diacetate-23-benzoate (**18**) and (22*R*)-22-hydroxy-24-nor-5β-cholan-3α,6α-diyl diacetate-23-benzoate (**19**).

¹H-NMR

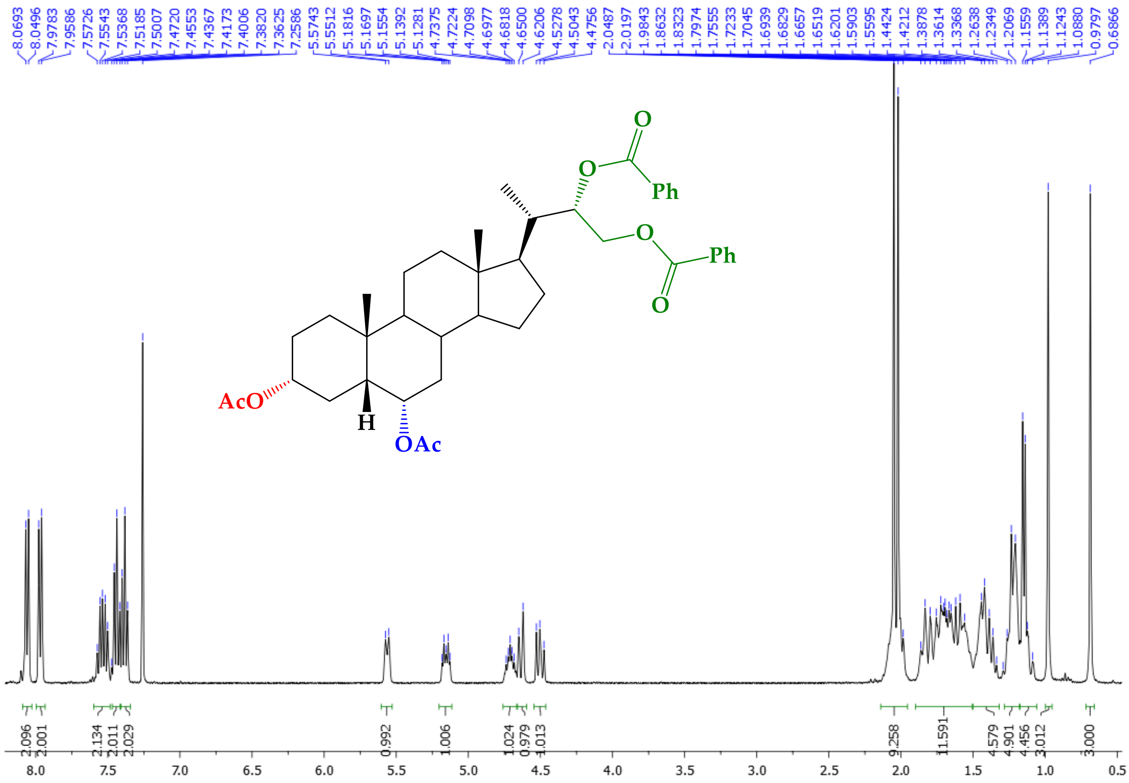

¹³C-NMR

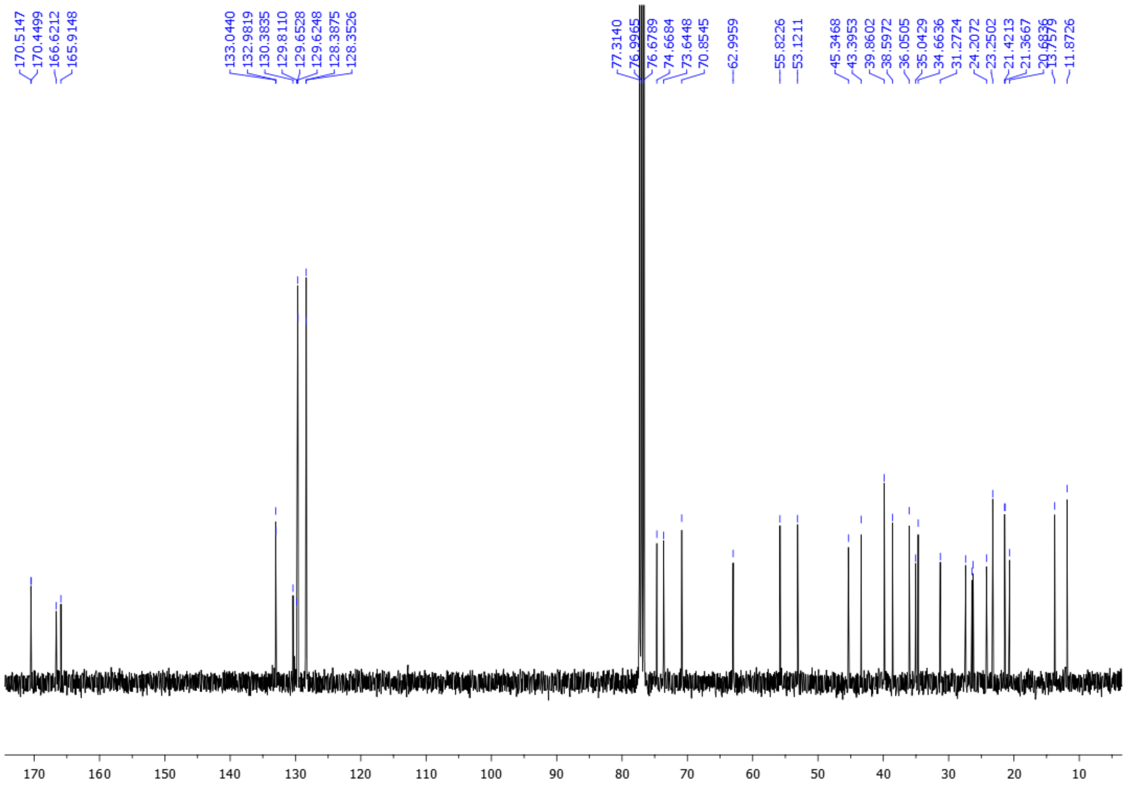

Figure S4: NMR spectra of (22*S*)-24-nor-5β-cholan-3α,6α-diyl diacetate-22,23-diyl dibenzoate (20)
